# Fabrication of Oil-Absorbing Porous Sponges via 3D Electrospinning of Recycled Expanded Polystyrene with Functional Additive

**DOI:** 10.3390/polym16233322

**Published:** 2024-11-27

**Authors:** Taegyun Kim, Seung Min Kang, Kanghyun Kim, Geon Hwee Kim

**Affiliations:** 1 Department of Mechanical Engineering, Chungbuk National University (CBNU), 1 Chungdae-ro, Seowon-gu, Cheongju-si 28644, Republic of Korea; 2 Department of Mechanical Engineering, Pohang University of Science and Technology (POSTECH), 77 Cheongam-Ro, Nam-Gu, Pohang 37673, Republic of Korea

**Keywords:** three-dimensional electrospinning, hydrophobic, oleophilic, expanded polystyrene (EPS), oil–water separation, recycling

## Abstract

In this study, a three-dimensional (3D) porous sponge capable of oil–water separation was fabricated using recycled expanded polystyrene (EPS) through 3D electrospinning, by adding phosphoric acid to the electrospinning solution. The fabrication process was a rapid and efficient single-step process to produce the 3D sponge. In addition, the additive’s concentration was also optimized for oil absorption. The fabricated EPS sponge was highly effective in oil–water separation due to its excellent hydrophobic and oleophilic properties. This demonstrates its potential as a sustainable and efficient absorbent to address ongoing oil pollution issues. Moreover, the performance of the recycled EPS sponge was found to be comparable to that of sponges made from virgin polystyrene, suggesting the feasibility of using recycled materials for the production of high-value products. This research presents an efficient method for fabricating 3D sponges from recycled materials, contributing to environmental protection and resource recycling.

## 1. Introduction

The global concern over plastic and polymer waste has significantly increased in recent years [1,2]. Over the past 60 years, approximately 60% of the 8.3 billion tons of plastic produced globally has been discarded [3]. Expanded polystyrene (EPS), commonly known as Styrofoam, has seen a rise in production due to its light weight and excellent insulation, soundproofing, and water resistance properties. This has consequently led to its increased accumulation in the environment [4,5,6]. The unique foamed structure of EPS makes it prone to fragmentation, contributing to microplastic pollution. Moreover, its non-biodegradable nature calls for urgent solutions to manage the waste it generates [3]. Therefore, there has been growing interest and research in the recycling of polymer waste to create high-value products. Recycling methods for polymer waste include pyrolysis [7,8,9,10], gasification [11,12,13], depolymerization [14,15,16] and mechanical (physical) recycling, which does not involve chemical reactions [17,18]. However, these methods tend to be costly, complex, and possibly produce harmful gases [19]. Therefore, recent research has focused on electrospinning technology, which is cost-effective, efficient, and versatile, making it applicable to a wide range of polymer wastes. Electrospinning is an efficient technique for producing long fibers of several nanometers from a polymer solution dissolved in a solvent, which can be used to fabricate filters, sensors, coatings, cotton, yarn, etc [20,21,22,23]. Applying a strong electric field to a polymer solution in a syringe creates a Taylor cone at the tip of the syringe, from which micro-/nanoscale fibers are produced on a charged collector. By adjusting the parameters of the process and the properties of the polymer solution, a wide range of control over the fabricated structures is possible. Nano-/microscale fibers are used in various research fields such as circuits, sensors, heaters, films, and porous membranes for oil–water separation or air filters [24,25,26,27,28]. Recently, studies have been reported on the development of three-dimensional structures from existing two-dimensional structures for various applications of nanofibers produced by electrospinning [29]. The structures produced by 3D electrospinning have various advantages compared to the existing 2D electrospun shapes. Compared to 2D structures, 3D structures have a higher surface area and more pores, which are applied in research to improve electrochemical performance, and have higher thermal insulation and filtering capabilities [30,31]. They also have potential for use in tissue engineering and energy storage applications, including cell growth [32,33]. Methods for fabricating 3D structures via electrospinning have been reported, including gas foaming [34], direct electrospinning [35] and the freeze-drying of electrospun mats [36], as well as methods combined with 3D printing [37]. The above methods are characterized by the need for additional chemical reactions in addition to electrospinning such as specialized collector devices and complex processes or experiments. The addition of additives to the electrospinning solution is simple and promises to produce 3D electrospun structures at very high speeds [30]. This process can produce large quantities of 3D structures with very small amounts of additives, while maintaining the advantages of electrospinning in producing micro-/nanostructures with small amounts of polymer. Reported additives include phosphoric acid, sodium hydroxide, hydrochloric acid, Iron(III) sulfate, yttrium(III) nitrate hexahydrate, and phosphoric acid [30]. The additives act to modulate the electrostatic interactions between fibers in the electrospinning process and allow electrospinning to produce bulk forms of 3D structures [38]. It is possible to produce 3D structures in the form of ultra-porous sponges in a very short time, and it is possible to produce sponges of various shapes by adjusting the electrospinning parameters. An ultra-porous EPS sponge can selectively absorb oil from oil–water mixtures, which can be a new solution for oil–water separation adsorbents. Research on the mechanical extraction of oil with adsorbents composed of nanofibers or aerogels with hydrophobic and oleophilic properties has been attracting attention [39,40,41]. Due to continuous industrial development and oil spills, a large amount of oily wastewater is generated, which is not easily degraded and poses an environmental hazard to the aquatic ecosystem [42]. An efficient production process of oil–water separation adsorbents based on recycled materials can be a new solution to the above environmental problems.

In this paper, we developed a rapid and efficient single-step process for fabricating 3D sponges capable of oil–water separation using recycled expanded polystyrene (EPS) through 3D electrospinning. By incorporating a small amount of phosphoric acid into the recycled EPS electrospinning solution, we were able to produce highly porous 3D structures with excellent hydrophobic and oleophilic properties. We investigated the optimal fabrication conditions, including additive concentration, to maximize oil absorption capacity. The morphology and oil–water separation performance of the fabricated EPS sponges were thoroughly characterized and compared to sponges made from virgin polystyrene. Our results demonstrate that recycled EPS can be transformed into high-value products with comparable performance to those made from virgin materials. This research not only presents an innovative method for upcycling EPS waste but also contributes to addressing environmental concerns related to plastic pollution and oil spills.

## 2. Materials and Methods

### 2.1. Materials

In this study, Tetrahydrofuran (THF, anhydrous, 99.5%) and Dimethylformamide (DMF, special grade, 99.5%) were purchased from Samchun Chemicals (Seoul, Republic of Korea). Phosphoric acid (ACS regent ≥ 85 wt%), Polystyrene (Mw~192,000), and Polystyrene (Mw~280,000) were purchased from Sigma-Aldrich (Louis, MO, USA). The oils were canola and soybean oil purchased from the market and mineral oil (extra pure) was purchased from Samchun Chemicals (Republic of Korea). All reagents were used without purification, and the other chemicals were commercial grade.

### 2.2. Preparation of Recycled EPS Solution

A solvent mixture of THF and DMF was prepared in a 1:1 ratio. THF and DMF have been reported as solvents for dissolving pure polystyrene in many studies and are also used for dissolving recycled EPS [43]. Recycled packaging EPS was washed in DI water and Ethanol and dissolved in a mixture of THF and DMF until 20 wt%. As a control, solutions of pure polystyrene with molecular weights of 192 k and 280 k were prepared. Many conductive materials could be additives; however, phosphoric acid was chosen because it is non-metallic and non-strong, in line with the theme of this study—recycling. Finally, phosphoric acid was added to each solution as an additive. The amounts of additives were set at 0, 10, 20, and 30 uL per 10 mL of the mixture. Mixed solutions were allowed to dissolve completely for 4 h at room temperature in a vortex mixer.

### 2.3. Three-Dimensional Electrospinning

The electrospinning process was used to fabricate a 3D sponge. First, recycled EPS solutions containing different amounts of phosphoric acid were electrospun. Three-dimensional electrospinning with additives can also be achieved using a regular flat plate stage. In this experiment, however, the ring-shaped stage made of aluminum foil with a diameter of 4 cm and a height of 2 cm was used to control the size of the 3D structures and ensure production reproducibility [30]. For uniform electrospinning, the electrospinning syringe was moved 3 cm repeatedly at a speed of 5 mm/s and the process time was 5 min. The flow rate was controlled using a flat-end metal needle with an outer diameter of 0.642 mm and an inner diameter of 0.337 mm. The voltage was set at 12 kV and the flow rate of the syringe pump was set at 0.083 mL/min. The tip-to-collector distance was set at 12 cm to ensure stable sponge production, with humidity maintained at 32–37% and the temperature was 18–22 °C. Control experiments with 192 k and 280 k molecular weight were also conducted under the same conditions. The above process was used to fabricate 3D porous sponges made of recycled EPS and pure polystyrene.

### 2.4. Oil–Water Separation

**Figure 1:**
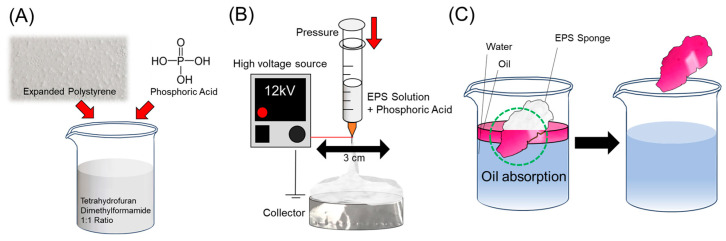
Schematic diagram of the fabrication process and application of the recycled EPS 3D structure. (**A**) EPS electrospinning solution composition with additives. (**B**) EPS 3D electrospinning with ring-shaped stage and moving syringe. (**C**) Application of manufactured EPS sponges as oil–water separation adsorbents.

The performance of the 3D sponge was evaluated for oil–water separation with respect to the oil absorption capacity and the separation efficiency. To verify the oil absorption performance of the sponge, the fabricated 3D sponge was immersed in the oil and the deionized (DI) water mixture, and was then compared by mass after complete drying. The weight of the fabricated sponge before and after oil absorption, the oil absorption capacity according to the unit g/g, was calculated as follows:

(1) $$Oil\;absorption\;capacity=\frac{m_{1}-m_{0}}{m_{0}}(g/g)$$

where *m*_0_ and *m*_1_ are the mass before and after absorption, respectively. In addition, the separation efficiency was calculated as follows:

(2) $$Separation\;efficiency=\frac{O_{1}}{O_{0}}\times 100\left(\%\right)$$

where *O*_0_ and *O*_1_ are the mass of oil in the initial mixture and the absorbed oil, respectively. of the entire fabrication and the oil–water separation process is described in Figure 1.

### 2.5. Characterization

High-resolution scanning electron microscopy (Gemini 560, ZEISS, Oberkochen, Germany) and scanning electron microscopy (SNE-ALPHA; SEC, Gyeonggi, Republic of Korea) were used to confirm the structure of the fabricated sponge. The water contact angle (WCA) was measured by Smartdrop Plus (Femtobiomed, Inc., Gyeonggi, Republic of Korea) using 5 μL of DI water. The infrared spectrum was measured using a Fourier-transform infrared spectrometer (Cary 670, Agilent, Santa Clara, CA, USA). The diameter and pore size of the fibers comprising the 3D structure were measured using ImageJ software (version 1.53e, Java 1.8.0_172, Wayne Rasband, U.S. National Institutes of Health, Bethesda, MD, USA).

## 3. Results and Discussion

### 3.1. Characteristic of 3D Electrospun Sponge

**Figure 2:**
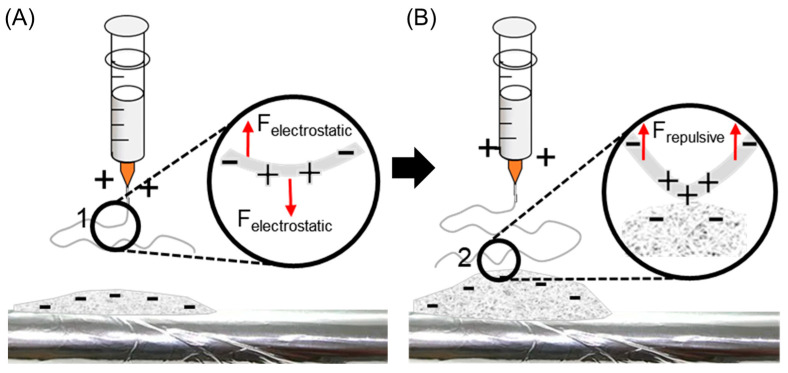
Mechanism of 3D electrospinning (**A**) near to and (**B**) far from the source.

The principle of 3D electrospinning by incorporating additives in polymer solutions has been investigated in several studies [44]. The formation of 3D structures by electrospinning is primarily governed by the polarization and electrostatic induction of the deposited fibers, combined with the rapid solidification of the electrospun fibers [45]. As fibers solidify, they form a self-standing structure. During the process, fiber deposited on the collector becomes negatively charged under the influence of a strong electric field. These negatively charged fibers attract positively charged solution jets from the nozzle while simultaneously repelling other negatively charged fibers. This electrostatic interaction prevents fiber compression and facilitates vertical stacking, resulting in a 3D morphology. The addition of H_3_PO_4_ enhances this process by increasing fiber polarizability, which amplifies the repulsive forces between the fibers and promotes vertical growth. This mechanism is illustrated in Figure 2. In Figure 2A, ionic charges within the solution jet near the nozzle are irregularly distributed due to polarization under the electric field. These charges experience electrostatic forces in a direction opposite to that of the electric field, aligning and directing the fibers toward the collector. Upon deposition, the top layer of fibers becomes negatively charged, creating a preferential deposition site for positively charged jets from the nozzle. As shown in Figure 2B, this negative charge at the top of deposited fibers induces polarization within incoming fibers. The resulting repulsive force on anions inside the jet prevents fiber compression and promotes vertical stacking. This interaction allows continuous fiber deposition along the nozzle’s movement path, forming a stable 3D structure. By combining charge induction, polarization effects, and enhanced fiber polarizability through H_3_PO_4_ addition, this process ensures stable vertical growth and prevents the collapse of the fibers into a dense 2D mat, enabling the formation of a robust 3D structure [38,46].

**Figure 3:**
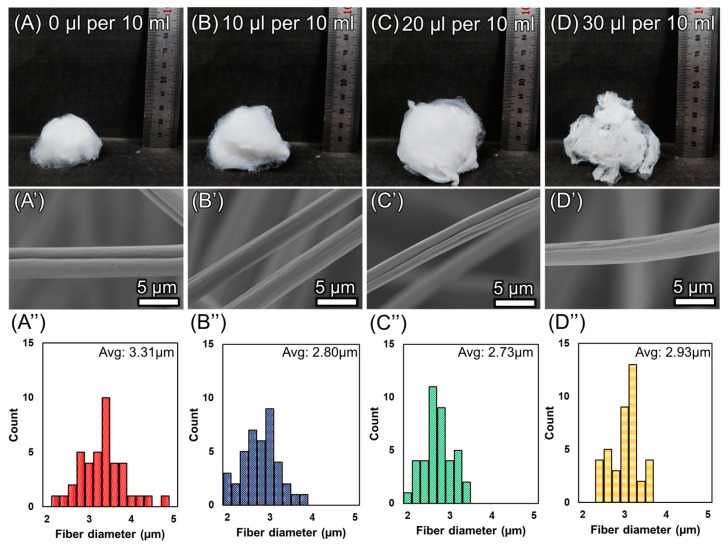
Morphology of 3D structural geometry as a function of additive concentration. (**A**) 0 μL per 10 mL of solution. (**B**) 10 μL per 10 mL of solution. (**C**) 20 μL per 10 mL of solution. (**D**) 30 μL per 10 mL of solution. (**A′**–**D′**) SEM images of the fibers composing the 3D structure. (**A″**–**D″**) Graph of fiber thickness distribution.

Figure 3 shows the morphological evolution of the 3D sponge structure as a function of phosphoric acid concentration in the EPS solution. To investigate the relationship between the conductivity and the 3D structure, the four concentrations of phosphoric acid additive are studied; 0, 10, 20, and 30 μL of phosphoric acid additive per 10 mL of solution. Figure 3A–D shows the 3D EPS sponge fabricated under the same conditions except for the amount of phosphoric acid solution. In the case of EPS, even in the absence of additives, a 3D-like structure can be obtained when using a ring-shaped collector (Figure 3A). Using a flat stage under the same conditions produced a small 3D structure. After the addition of phosphoric acid solution, sponges with a larger volume and surface area were produced under the same electrospinning conditions (Figure 3B). The largest volume and the fluffiest sponge morphology were produced when 20 μL of additive was added per 10 mL of solution (Figure 3C). For an additive amount of 30 μL per 10 mL, a 3D sponge with a locally dense morphology was produced (Figure 3D), resulting in a structure that lacks fluffiness, and exhibits reduced mechanical strength. Figure 3A′–D′ are representative of a single fiber of the sponge for each additive concentration. There is little difference in fiber thickness before and after additive mixing and by concentration, and the surface of the fiber is also not different. In addition, Figure 3A″–D″ show the thickness distribution of the fibers for each condition. All conditions showed fiber diameter distributions within the range of 2–5 μm, and the average thickness was around (near) 3 μm. Thus, the addition of phosphoric acid additives has minimal impact on the surface structure and thickness of the fibers, while it significantly influences the morphology of the 3D sponge.

**Figure 4:**
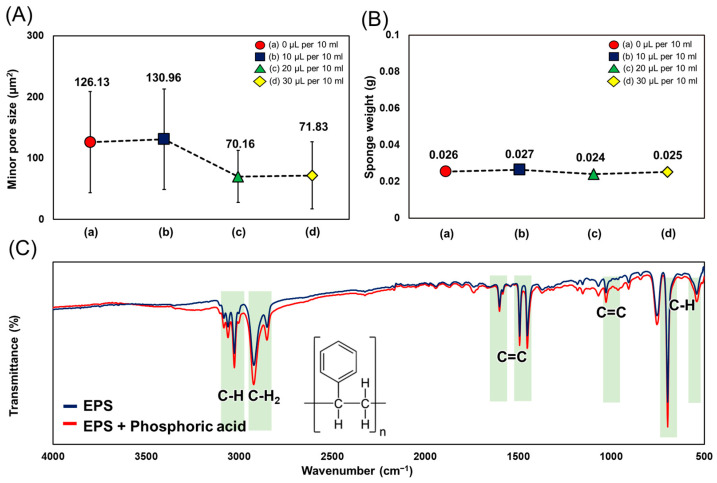
Comparison of (**A**) minor pore size. (**B**) The weight of the sponge with the additive at several concentrations. (**C**) The Fourier-transform infrared (FT-IR) spectrum of the 3D sponge in the presence of a phosphoric acid solution.

Figure 4 shows the characteristics of sponges (A)–(D) according to the additive concentration presented in Figure 3. Figure 4A shows the size of small pores between the fibers, which could block the hydrophilic liquid and hold the oil. For case (a), without any additives used, the pore size distribution ranged from 28 to 356 μm^2^ with a mean value of 126.13 ± 82.83 μm^2^. Case (b), with 10 μL of additive mixed per 10 mL of electrospinning solution, shows almost the same minor pore size distribution and mean as in case (a). When the amount of additive is greater than 20 μL per 10 mL, the average value of the minor pore size decreases to 70.16 ± 42.64 μm^2^, and the case (d) with 30 μL per 10 mL shows a similar pore size of 71.83 ± 54.85 μm^2^. Based on the morphologies of the sponge in Figure 3, it has been shown that case (c), which is the 20 μL per 10 mL condition, produced 3D sponges with the best for oil–water separation, with a large volume, uniformity, and the smallest pore size. Figure 4B shows that sponges of all conditions have approximately the same weight, which indicates only porosity was controlled for. Figure 4C shows the results of the Fourier-transform infrared spectroscopy (FT-IR) of the sponge with and without additives. The chemical structure of the EPS itself remains unchanged by any chemical reaction or chemical reason, and the additives influence the electrostatic properties of the electrospun fibers without altering the chemical structure.

### 3.2. Comparison of Pure Polystyrene and EPS Sponge

**Figure 5:**
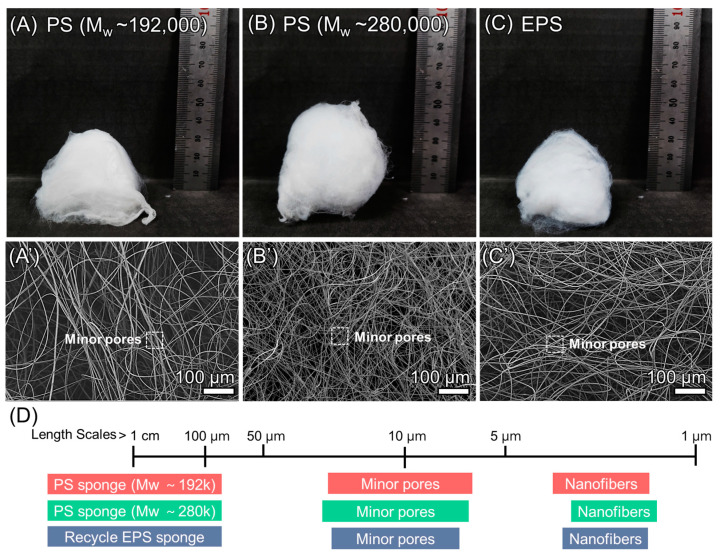
Comparison of two pure polystyrenes and EPS. Images of the fabricated 3D structure made of (**A**) polystyrene (M_w_~192,000), (**B**) polystyrene (M_w_~280,000), (**C**) EPS. (**A′**–**C′**) SEM images for pore size analysis. (**D**) Scale graph for each structure.

In Section 3.1, the optimal conditions for the application of 3D electrospinning with EPS as an oil–water separation adsorbent were verified. The highest surface area EPS sponge was produced under the condition of adding 20 μL of phosphoric acid solution per 10 mL of electrospun solution. Figure 5 shows the comparison of pure polystyrene with Mw~192,000, polystyrene with Mw~280,000, and EPS sponges prepared under optimal conditions. The performance difference between the two pure polystyrene and EPS sponges is verified under the conditions established in Section 2.3. The shape of the electrospun sponges exhibits no significant differences, as shown in Figure 5A–C. Furthermore, the fiber distribution also is shown in a similar way as shown in Figure 5A′–C′. Figure 5D organizes a scale for each sponge. This shows that the structure is multiscale, which makes it possible to absorb the oil. The minor pore in Figure 5D is measured based on the small pore defined by the closest fiber layers. All three conditions exhibited minor pores with diameters ranging from 6 to 28 μm and a scale of nanofibers ranging from 2 to 4 μm thick. Therefore, the sponge produced from recycled EPS can produce a structure like that of a sponge produced from pure EPS and has the potential to be used as a 3D sponge fabrication material that can replace polystyrene.

### 3.3. The Oleophilic and Hydrophobic Properties of the 3D Structure

**Figure 6:**
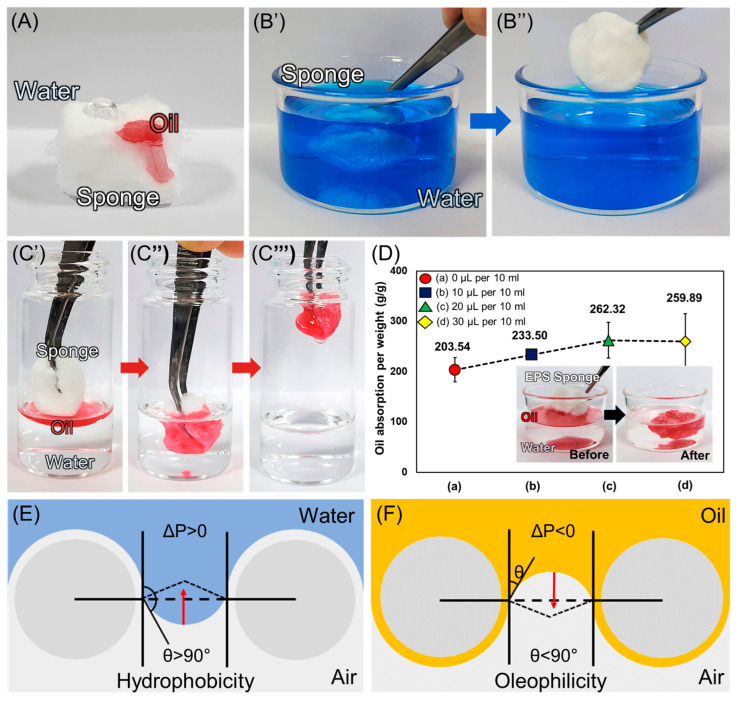
(**A**) The oleophilic and hydrophobic properties of the fabricated 3D sponge. (**B′**,**B″**) The oil absorption performance of the fabricated 3D sponge. (**C′**–**C‴**) The hydrophobicity of the fabricated 3D sponge. (**D**) Oil absorption per unit. (**E**) A schematic of the water wetting mode of the hydrophobic membrane. (**F**) A schematic of the oil wetting mode of the oleophilic membrane.

EPS is mainly composed of polystyrene and has inherently hydrophobic and oleophilic properties. As shown in Figure 6A, the EPS sponge converged to a water contact angle of 121.9° with DI water and 0° with canola oil. In Figure 6B′,B″, the sponge does not become wet even when fully immersed in DI water mixed with the blue water-based colorant. However, Figure 6C′–C‴ depict that the sponge completely absorbed the oil in a mixture of oil and water. These results indicate that the fabricated sponge is effective as an oil adsorbent for oil–water separation. Figure 6D shows the oil absorption per unit weight of the sponge produced as a function of the amount of additive, which is one of the crucial properties for oil–water separation. The 20 μL per 10 mL condition with the largest volume, uniformity, and smallest minor pore size was found to have the highest oil absorption per unit weight of 262.32 g/g. When it comes to the oil–water separation performance, the pore size defined by the nanofiber networks is the most critical. It determined the breakthrough pressure, which is the maximum pressure required for a liquid to penetrate the pores of the membrane. It is represented by the Young–Laplace equation as follows [47,48].

(3) $$∆P=-\left(\frac{2\gamma_{L}cos\theta }{r_{p}}\right)$$

In the above equation, Δ*P* is the breakthrough pressure, *γ_L_* is the interfacial surface tension of oil and water, *θ* is the contact angle of water and oil, and *r_p_* is the radius of the pore. Polystyrene is a hydrophobic, oleophilic material, and when in contact with water, the contact angle is greater than 90° and the breakthrough pressure is greater than 0. Therefore, it exhibits a negative capillary effect, and water is not permeable (Figure 6E). For oil, the oil contact angle is lower than 90° and the breakthrough pressure is less than 0. Therefore, the membrane cannot support the pressure, and oil can permeate the surface through a positive capillary effect (Figure 6F) [47,49,50]. In addition, the smaller the pore size of the nanomembrane, the greater the breakthrough pressure on the hydrophobic surface, which can better repel water, and the smaller the breakthrough pressure on the oleophilic surface, which can easily permit oil to pass. Based on the above theory, the fabricated polystyrene-based 3D sponge can efficiently separate water and oil, and the pore size can be controlled by adjusting the volume of additives, thereby enhancing the separation efficiency.

### 3.4. Oil–Water Separation Performance

**Figure 7:**
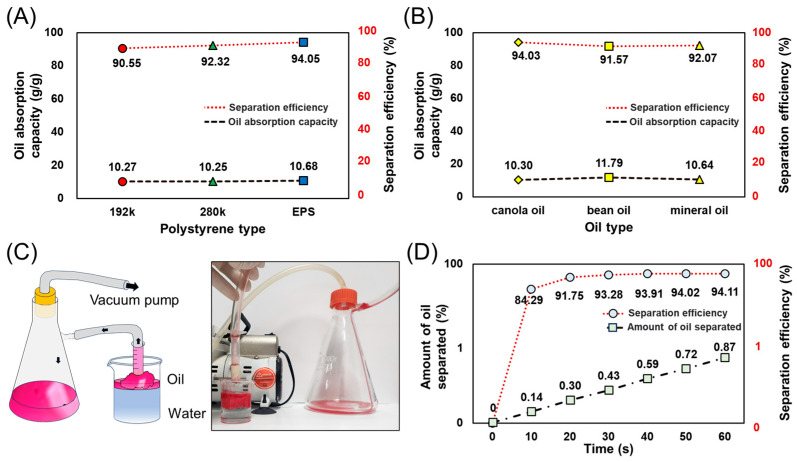
The experimental results of oil absorption capacity and images of the experimental apparatus. (**A**) A graph of the oil absorption capacity and separation efficiency of the three polystyrenes (M_w_~192,000, M_w_~280,000, and EPS). (**B**) A graph of the oil absorption capacity and separation efficiency of the three different oils (canola oil, bean oil, and mineral oil) with the EPS sponge. (**C**) A schematic and images of the device for continuous oil–water separation. (**D**) A graph of oil absorption by the sponge and the amount of oil separated over time.

The oil–water separation performance was evaluated based on the morphology and the wettability of the fabricated sponges. Figure 7A shows the oil–water adsorption performance of the three sponges prepared under the condition of 20 μL of addition per 10 mL of phosphoric acid solution. All three conditions showed a similar oil absorption capacity of slightly over 10 g/g and a separation efficiency of over 90%. Figure 7B shows the separation efficiency of the EPS sponge for various oils: canola oil, bean oil, and mineral oil. The oil absorption capacity for all the oils is over 10 g/g and the separation efficiency is over 90%; as same as the previous result. However, the usage is restricted by some solvents which make it possible to dissolve the sponge, such as chloroform and DCM. Figure 7C shows a schematic diagram and photo of the device for continuous oil separation and reproduction utilizing the fabricated EPS sponge. A vacuum pump, flask, and syringe were utilized to reproduce the oil, and a metal mesh was used to prevent the sponges from separating altogether. Figure 7D describes the time-dependent oil absorption and separation efficiency of the EPS sponge with the apparatus. It absorbed approximately 84% of the oil in the oil–water mixture in 10 s, 91% in 20 s, and 94% within 30 s. The fabricated reproduction device showed a reproduction yield of 0.87 g of oil in 60 s. This demonstrated the rapid and highly efficient oil adsorption performance of the sponge and the possibility of continuous separation and oil reproduction. This highlights its potential as a practical and effective primary approach for addressing oil contamination in oil–water mixtures.

To evaluate the oil–water separation performance of the prepared EPS sponge, the oil absorption capacity was compared with various oil–water separation sponges reported in previous studies (Table 1). The oil absorption capacities of the sponges and adsorbents reported in previous studies ranged from 6.74 g/g to 60 g/g. The EPS sponge produced by a single material and process exhibited an oil absorption capacity of approximately 11 g/g, demonstrating the feasibility of producing a highly efficient sponge from recycled materials. In particular, the oil absorption performance was similar to that of sponges made from natural materials or biomass, such as wood sponges, hybrid PET aerogels, rice straw polyurethane foams, and the FXBW-PU sponge. This suggests that waste EPS can be recycled to produce high-performance adsorbents. On the other hand, sponges using composite materials, such as magnetic polyurethane sponges or polylactic acid-Calotropis gigantea foams, or sponges with post-treatment processes, showed relatively high oil absorption capacities. Therefore, it is anticipated that the oil absorption performance of EPS sponges can be further maximized by compounding with other materials or surface modification. The comparative results presented above demonstrate that EPS sponges fabricated by 3D electrospinning exhibit comparable performance to conventional adsorbents for oil–water separation. The significance of this study is that high-performance sponges can be prepared by a simple process utilizing recycled materials. In addition to evaluating the adsorption performance for various pollutants, further research on long-term stability and reusability will enhance the potential for practical applications.

## 4. Conclusions

This study demonstrates a sustainable and efficient approach to upcycling waste EPS into high-performance 3D sponges for oil–water separation. By optimizing the 3D electrospinning process with phosphoric acid as an additive, we have developed a rapid, single-step fabrication method that produces sponges with superior oil absorption capacity and separation efficiency, comparable to those made from virgin polystyrene. Three-dimensional electrospinning using phosphoric acid additives was more rapid and efficient than the existing method, which requires complex processes and additional devices. Because the concentration of phosphoric acid is the most critical variable in terms of the morphology, it was optimized to 20 μL in 10 mL with the smallest minor pore size of 70.16 μm^2^ and the largest oil absorption capacity of 262.32 g/g. It was also confirmed that recycled EPS exhibited similar morphology, physical properties, and oil–water separation performance to pure polystyrene. The EPS sponge demonstrated a high oil absorption capacity of over 10 g/g and an oil–water separation over 90%, attributed to its large surface area, small pore size, and excellent hydrophobic and oleophilic properties, comparable to those of pure polystyrene sponges. The EPS sponge has potential not only as an oil–water separation adsorbent, but also in diverse applications such as high-performance filters, tissue engineering, and energy storage devices. Furthermore, this suggests the feasibility of utilizing recycled materials as high value-added products using recycled polymers, beyond environmental protection and resource recycling.

## Figures and Tables

**Table 1:** Reported oil absorption capacity of other oil–water separation sponges.

Materials	Oil Absorption Capacity (g/g)	References
Wood sponge	12.72	[51]
Hybrid PET aerogel	13–27	[52]
Magnetic polyurethane sponge	16–60	[53]
Rice straw polyurethane foams	10.75–15.09	[54]
Polylactic acid nanoporous fibrous membranes	6.74–42.38	[55]
FXBW-PU sponge	11.79–26.59	[56]
Polylactic acid–Calotropis gigantea foam	22.80–48.30	[57]
Expanded polystyrene sponge	10.30–11.79	This work

## Data Availability

The datasets generated during and/or analyzed during the current study are available from the corresponding author on reasonable request.
